# Quantile-based effect of energy, transport, and total environmental tax on ecological footprint in EU5 countries

**DOI:** 10.1007/s11356-024-32214-3

**Published:** 2024-02-17

**Authors:** Mustafa Tevfik Kartal

**Affiliations:** 1 Department of Banking and Finance, European University of Lefke, Lefke, Northern Cyprus Türkiye; 2 Strategic Planning, Financial Reporting, and Investor Relations Directorate, Borsa Istanbul, Istanbul, Türkiye; 3 Adnan Kassar School of Business, Lebanese American University, Beirut, Lebanon; 4 Clinic of Economics, Azerbaijan State University of Economics (UNEC), Baku, Azerbaijan

**Keywords:** Environmental tax, Ecological footprint, EU5 countries, Quantile-based methods

## Abstract

Considering a vast majority of application areas, the study investigates how environmental tax (ET) affects ecological footprint. In this context, the study examines the European Union Five (EU5) countries, considers ecological footprint (EF) as the proxy of the environment, uses ET as tax-based environmental measures by making both disaggregated (i.e., energy and transport) and aggregated level analysis, and performs novel nonlinear quantile-based approaches for the period from 1995/Q1 to 2021/Q4. The outcomes show that on EF (i) energy-related ET has only a declining effect at lower and middle quantiles in Germany and at lower quantiles in Italy, whereas it does not have a curbing effect in other countries; (ii) transport-related ET is not effective on EF in any country, which means that it does not have a curbing effect; (iii) total ET has a decreasing effect in only Germany; and (iv) the alternative method validates the robustness. Thus, the study demonstrates the changing effect of ET across countries, quantiles, and ET types in curbing EF. Hence, it can be suggested that Germany can go on relying further on energy-related ET practices to decrease EF, whereas there is a long way for the remaining EU5 countries as well as transport-related ET in curbing EF.

## Introduction

All humanity has been facing a recent climate change crisis recently. The main reason for this negative challenge is anthropogenic, which results from human activities. Because the negative effects of climate-related problems on humanity have been increasing, public awareness has been developing day by day to combat such negative progress (Zhan et al. 2021; Ayhan et al. 2023; Bekun 2024; Pata et al. 2024). In this context, all related parties including countries, policymakers, and scholars have been increasing their research on the causes of climate-related problems and the solution ways to these determinations (Kartal et al. 2023).

According to the literature, a variety of factors has been causing climate-related problems, especially an increase in CO_2_ emissions and EF. Among various causes, such causes as higher economic growth, intensive fossil fuel energy use, and much more end-user consumption have a leading role because they have a significant increasing effect on environmental degradation (Anser et al. 2023; Chu et al. 2023; Nwani et al. 2023; Pata et al. 2023a). Therefore, policymakers aim to decrease such negative progress by taking various measures. In addition to other regulatory regulations, putting ET into effect is evaluated as a significant tool to combat with environmental pollution (Pigou 1920; Chien et al. 2021a, 2021b; Ullah et al. 2023). In this way, countries aim to achieve some SDGs, especially SDG-7 and SDG-13, which are related to clean energy use and climate action. Thus, countries can have the opportunity to achieve eco-friendly economic growth without causing environmental pollution (Miao et al. 2022).

When environmental pollution and ET practices can be evaluated together, it can be seen that EU countries have a leading role in terms of their efforts to become carbon–neutral. Also, EU countries have had a better ET structure in various areas over the years (OECD 2023). Moreover, EU countries have a relatively bigger economic size among countries. So, the examination of EU countries in investigating the link between ET and environmental pollution can be an important example in understanding how ET affects curbing environmental pollution because EU countries have a lighthouse role for other countries in combating climate change problems as well.

**Fig. 1:**
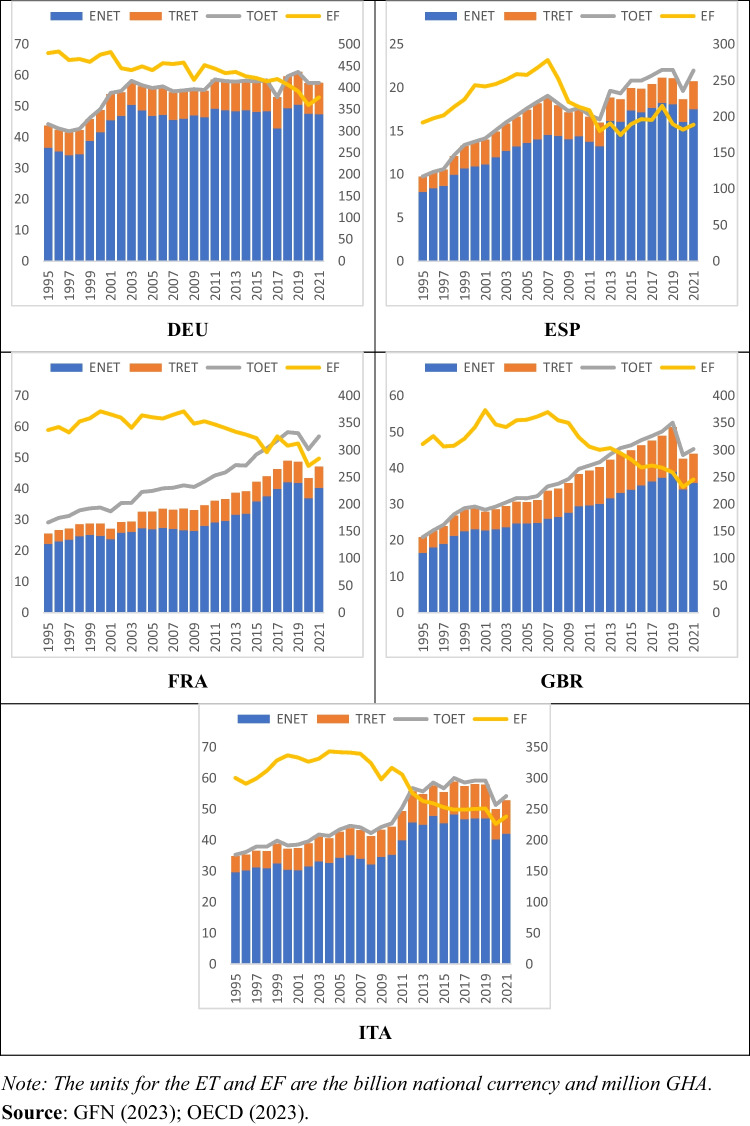
Development of ET and EF

On the other hand, while considering the causes of environmental pollution and applying ET in various areas, it is also crucial that the potential declining effects of ET on the areas, where ET is applied, should be considered. According to OECD (2023), ET can be forced into various environmental domains (e.g., fossil fuels, renewable energy, air pollution, and climate change) and it can be listed under a total of four groups (i.e., energy, transport, pollution, and resources). Accordingly, considering the sub-types of ET in such areas is important. So, focusing on disaggregated level ET rather than handling ET as a whole (i.e., total ET) can be the right approach. So, Fig. 1 demonstrates the progress of ET and environmental pollution proxied by the EF in EU5 countries (Germany, DEU; Spain, ESP; France, FRA; Great Britain, GBR; and Italy, ITA).

As presented in Fig. 1, EU5 countries have had an ET structure over the year since 1995. Also, they have the most recent data reaching to the year of 2021. Among all, DEU and FRA have a leading role in terms of total ET, whereas IT, GBR, and ESP have the following order. Also, ENET has the highest share in total ET in all countries, followed by TRET. Besides, when the environmental pollution is examined, it can be seen that there is a relative decrease in recent years with regard to previous years; however, this decrease is not so sharp to ensure carbon-neutrality targets of EU 5 countries.

Because ET is an option for countries to combat with environmental pollution, various countries have been imposing ET into various areas. Accordingly, a growing number of studies have been dealing with ET in searching the progress of environmental pollution. For example, Irz et al. (2019) investigate some EU countries (i.e., Denmark, Finland, and France); He et al. (2021) analyze China and Malaysia; Depren et al. (2023) and Sharif et al. (2023) examine Nordic countries; Hao et al. (2021), Dogan et al. (2022), and Onwe et al. (2023) uncover G7 countries. Among all, based on the best knowledge, the literature does not include any study, which focuses on EU5 countries, makes a country-based analysis to take country-based potential differences into account, and realizes both disaggregated and aggregated level ET analysis together. Hence, in the researcher’s belief, the literature has a gap from this point perspective.

Considering the gap defined, this research analyzes EU5 countries. In this context, the research prefers to use EF as the environmental pollution proxy instead of CO_2_ emissions to consider various sides of environmental pollution (i.e., water and soil, in addition to air) and apply analysis based on each country to take potential differences among the countries into account, realizes the analysis on disaggregated and aggregated level ET data, and applies novel nonlinear quantile-based approaches from 1995/Q1 to 2021/Q4. Thus, the study investigates the following: (i) How ET affects EF? (ii) Does the effect of ET differ according to ET types (i.e., energy, transport, and total)? (iii) Does the power effect of ET on EF vary based on quantiles and countries? In searching for answers to these research questions, the study determines that (i) energy-related ET has only a declining effect at lower and middle quantiles in Germany and at lower quantiles in Italy; (ii) transport-related ET is not effective on EF in any country, which means that it does not have a curbing effect; and (iii) total ET has a decreasing effect in only Germany. Hence, the study provides some contributions as follows: (i) the study is the leading study for EU5 countries in investigating the link between ET at both disaggregated and aggregated levels and EF by realizing a country-based analysis; (ii) differentiating from the most of present studies, the study uses EF as a more comprehensive environmental pollution indicator with regard to CO_2_ emissions because it considers also water and soil pollution; (iii) the research applies novel nonlinear quantile-based approaches to investigate the effect of ET on EF across quantiles.

The second part reviews the literature. The third part presents the methods. The fourth part presents the outcomes. The last part concludes with policy endeavors and further research directions.

## Literature review

In the present literature, a variety of proxy indicators for environmental pollution has been used by researchers. Many earlier studies have used CO_2_ emissions as a proxy of environmental pollution indicators (e.g., Ulucak et al. 2020; Adebayo and Kartal 2023; Hussain et al. 2023; Kartal et al. 2023; Wang et al. 2023).

Although it can be sensible for scholars to use CO_2_ emissions at the initial step of environmental examination due to the data unavailability for other proxies, it is not now logical because there is data on EF, which enables researchers to consider also water and soil pollution, in addition to air pollution. Accordingly, many later studies have used EF as the proxy for environmental pollution. For instance, Kartal and Pata (2023) uncover the environment in China by using EF. Pata et al. (2023b) analyze China’s emissions by considering EF. Also, Pata et al. (2023c) examine Turkey’s environment relying on EF. By taking into account that EF is a much more comprehensive proxy for environmental pollution, the study uses EF as the proxy for the environment.

In the case of ET application, the literature includes also various studies. According to the research outcomes, some studies have defined that ET as a beneficial tool in curbing environmental pollution (e.g., Hussain et al. 2023; Kirikkaleli 2023). However, the literature does not have a consensus on the effect of ET on environmental pollution because some other studies have concluded that ET is inefficient in preventing environmental pollution (e.g., Rybak et al. 2022; Xie and Jamaani 2022).

To examine the literature from the point of scope view, it can be stated that some country groups and countries have been examined much more frequently than others. Among all, BRICS, OECD, and Nordic countries have been more frequently analyzed by scholars in the present literature (e.g., Ulucak et al. 2020; Depren et al. 2023). On the other hand, some countries, such as Malaysia and Latvia, have been included much less in empirical research (e.g., Loganathan et al. 2020; Brizga et al. 2022). Generally, aggregated level ET data has been used in the studies, whereas a few studies have preferred to use disaggregated level ET data (e.g., Loganathan et al. 2020; Rybak et al. 2022 consider ENET; Meireles et al. 2021; Brizga et al. 2022 use TRET).

From the empirical analysis perspective, the current studies have also applied a variety of econometric approaches in empirical investigations. These studies have mainly applied estimation models, such as autoregressive distributed lag, based on mean points. However, such methods do not consider that the effect of ET on the environment can be varying according to levels (i.e., quantiles) because they provide estimation outcomes for mid-point.

To sum up, although there are various studies in the literature, there has not been a consensus about ET's effect on the environment. Also, there is still a gap in the literature, which is that no study examines EU5 countries by applying a country-based analysis and using both disaggregated and aggregated level ET data together.

## Methods

### Data

The study analyzes the effect of ET on EF in EU5 countries. In doing so, data on ET and EF is gathered from OECD (2023) and GFN (2023), in order. After gathering data from the sources, the dataset is firstly transformed into a quarterly period applying a quadratic sum approach (Ulussever et al. 2023), and then, logarithmic difference series are obtained (Ali et al. 2023). Thus, the study uses the dataset between 1995/Q1 and 2021/Q4.

Table 1 explains the details of the variables.

**Table 1:** Variables

Symbol	Definition	Unit	Source
EF	Ecological footprint	Million Global Hectares	GFN (2023)
ENET	Energy-related ET	Million National Currency	OECD (2023)
TRET	Transport-related ET		
TOET	Total ET		

### Empirical approach

**Fig. 2:**
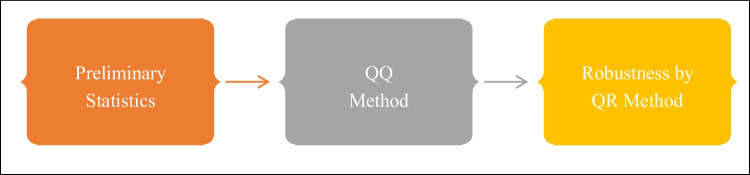
Empirical methodology

The study applies a three-step empirical methodology in empirical analysis to investigate the ET effect on EF in EU5 countries at both disaggregated and aggregated levels as shown in Fig. 2.

In the first step, descriptive statistics and correlations are first examined. Later, the nonlinearity condition of the variables is analyzed by using by the BDS test (Broock et al. 1996) test. In the second step, the QQ method is applied to investigate the ET effect on EF across quantiles (Sim and Zhou 2015). In the last step, the QR method is performed to validate the robustness of the QQ outcomes (Koenker and Bassett 1978). By following up on this empirical methodology, the study makes a deepened analysis of the effect of EF on EF across various levels (quantiles) rather than a mid-point.

## Empirical outcomes

### Preliminary statistics

In the first step, the study analyzes the preliminary statistics of the dataset. In this context, Table 2 presents the descriptive statistics.

**Table 2:** Descriptive statistics

Country	Variable	Mean	Max	Min	SD	Skewness	Kurtosis	JB	JB Prob	Unit
DEU	ENET	11,320.34	12,703.34	8465.96	1233.82	− 1.19	3.08	25.48	0.0000	Million Euro
	TRET	2173.49	2647.71	1700.63	284.98	− 0.11	1.68	8.08	0.0176	Million Euro
	TOET	13,549.80	15,353.05	10,481.55	1402.31	− 1.13	2.94	23.09	0.0000	Million Euro
	EF	109.86	121.76	89.48	7.70	− 0.76	3.28	10.72	0.0047	Million GHA
ESP	ENET	3450.44	4719.54	1949.30	755.80	− 0.28	2.15	4.66	0.0974	Million Euro
	TRET	718.57	1098.06	432.80	152.61	0.41	3.32	3.42	0.1808	Million Euro
	TOET	4273.00	5981.20	2387.31	908.91	− 0.46	2.33	5.74	0.0566	Million Euro
	EF	54.37	70.15	43.41	7.52	0.44	1.92	8.81	0.0122	Million GHA
FRA	ENET	7465.46	10,836.09	5484.78	1537.57	0.83	2.35	14.20	0.0008	Million Euro
	TRET	1383.82	1821.92	673.75	371.01	− 0.53	1.55	14.53	0.0007	Million Euro
	TOET	10,633.40	15,134.16	7045.55	2241.36	0.35	1.95	7.19	0.0275	Million Euro
	EF	84.74	93.42	66.82	6.57	− 0.99	3.41	18.50	0.0001	Million GHA
GBR	ENET	6954.11	9,858.07	4010.57	1556.40	0.11	1.97	5.03	0.0810	Million Sterling
	TRET	1969.37	3,190.57	1046.70	619.55	0.24	1.65	9.28	0.0097	Million Sterling
	TOET	9178.19	13,363.48	5056.66	2237.83	0.05	1.85	6.05	0.0485	Million Sterling
	EF	78.52	93.95	57.51	9.79	− 0.33	2.15	5.21	0.0739	Million GHA
ITA	ENET	9400.18	12,153.86	7253.37	1671.57	0.45	1.54	13.10	0.0014	Million Euro
	TRET	2127.89	2869.52	1267.65	465.14	− 0.53	2.13	8.52	0.0141	Million Euro
	TOET	11,787.84	15,124.57	8642.96	2104.07	0.30	1.54	11.13	0.0038	Million Euro
	EF	74.00	86.25	56.03	9.42	− 0.31	1.66	9.87	0.0072	Million GHA

Min minimum, Max maximum, SD standard deviation, JB Jarque–Bera

According to Table 2, ET has a higher volatility than EF in all countries. On the other hand, TOET is much more volatile than ENET and TRET. Moreover, based on the JB outcomes, it can be seen that all variables have a nonnormal distribution at a 10% significance level.

Following descriptive statistics, Table 3 demonstrates correlations.

**Table 3:** Correlations

Country	Variable	ENET	TRET	TOET	EF
DEU	ENET	1.00			
	TRET	0.05	1.00		
	TOET	0.98	0.24	1.00	
	EF	− 0.07	0.42	0.03	1.00
ESP	ENET	1.00			
	TRET	0.59	1.00		
	TOET	0.98	0.72	1.00	
	EF	0.60	0.45	0.62	1.00
FRA	ENET	1.00			
	TRET	0.20	1.00		
	TOET	0.93	0.53	1.00	
	EF	0.55	0.32	0.61	1.00
GBR	ENET	1.00			
	TRET	0.86	1.00		
	TOET	0.98	0.94	1.00	
	EF	0.45	0.27	0.39	1.00
ITA	ENET	1.00			
	TRET	0.58	1.00		
	TOET	0.99	0.66	1.00	
	EF	0.15	0.56	0.23	1.00

According to Table 3, EF has only a negative relationship with ENET in DEU, whereas its relationship with ENET in other countries is positive. Also, EF has a positive relationship with TRET in all countries. Then, Table 4 presents the nonlinearity test outcomes.

**Table 4:** Nonlinearity test outcomes

Country	Variable	DM2	DM3	DM4	DM5	Decision
DEU	ENET	0.0000	0.0000	0.0000	0.0000	NL
	TRET	0.0000	0.0000	0.0000	0.0000	NL
	TOET	0.0000	0.0000	0.0000	0.0000	NL
	EF	0.0251	0.7784	0.2713	0.6366	M
ESP	ENET	0.0000	0.0008	0.0062	0.0000	NL
	TRET	0.0000	0.0000	0.0000	0.0000	NL
	TOET	0.0000	0.0036	0.1043	0.0026	M
	EF	0.0000	0.0000	0.0001	0.0000	NL
FRA	ENET	0.0004	0.0564	0.5582	0.1081	M
	TRET	0.0000	0.0000	0.0000	0.0000	NL
	TOET	0.0005	0.0643	0.5058	0.0589	M
	EF	0.1606	0.7716	0.1945	0.9772	L
GBR	ENET	0.9178	0.8877	0.8635	0.8420	L
	TRET	0.0000	0.0000	0.0000	0.0000	NL
	TOET	0.9178	0.8877	0.8635	0.8585	L
	EF	0.0000	0.0007	0.0453	0.0353	NL
ITA	ENET	0.0400	0.7065	0.6086	0.3755	M
	TRET	0.0000	0.0000	0.0032	0.0011	NL
	TOET	0.0308	0.6424	0.6809	0.3611	M
	EF	0.0000	0.0000	0.0000	0.0000	NL

Values indicate probability values. DM, L, NL, and M denote the dimension, linear, nonlinear, and mixed, in order

According to Table 4, the variables have mixed structure in terms of their structure across dimensions. Among all, some of them have nonlinear structures, whereas a few of them are linear. Hence, most of the variables have a nonnormal distribution as well as a nonlinear characteristic rather than following a normal distribution and having a linear structure, which implies that there are significant variations in the variables. Based on these determinations, the study performs novel nonlinear quantile-based approaches for empirical analyses.

### QQ outcomes

**Fig. 3:**
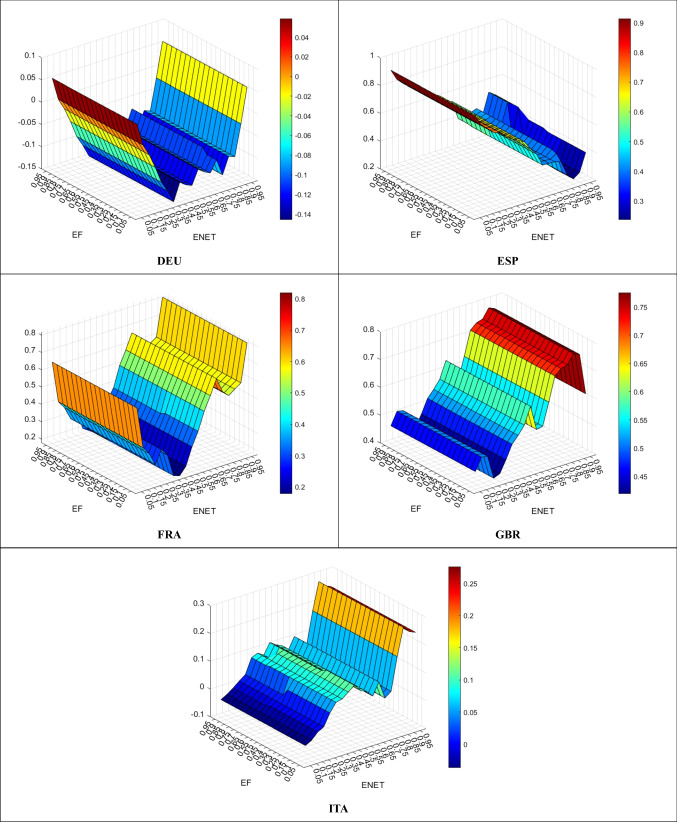
ENET effect on EF

After examining preliminary statistics, the study applies the QQ method to investigate the effect of ET on EF across quantiles. So, Fig. 3 shows the effect of ENET on EF.

In DEU, ENET has a declining effect on EF across all quantiles except for some lower (0.05–0.10) and higher (0.95) ones. In the ITA case, ENET has a declining effect on EF only at some lower quantiles (0.05–0.15). In the other countries (i.e., ESP, FRA, and GBR), ENET has a fully increasing effect on EF. At higher quantiles, the effect of ENET is highly increasing in FRA and GBR, whereas the increasing effect becomes much weaker in ESP.

Overall, while ENRET is highly efficient on environmental pollution in DEU, it is a bit beneficial in ITA. On the other hand, an increase in ENRET does not help curb environmental pollution in ESP, FRA, and GBR.

**Fig. 4:**
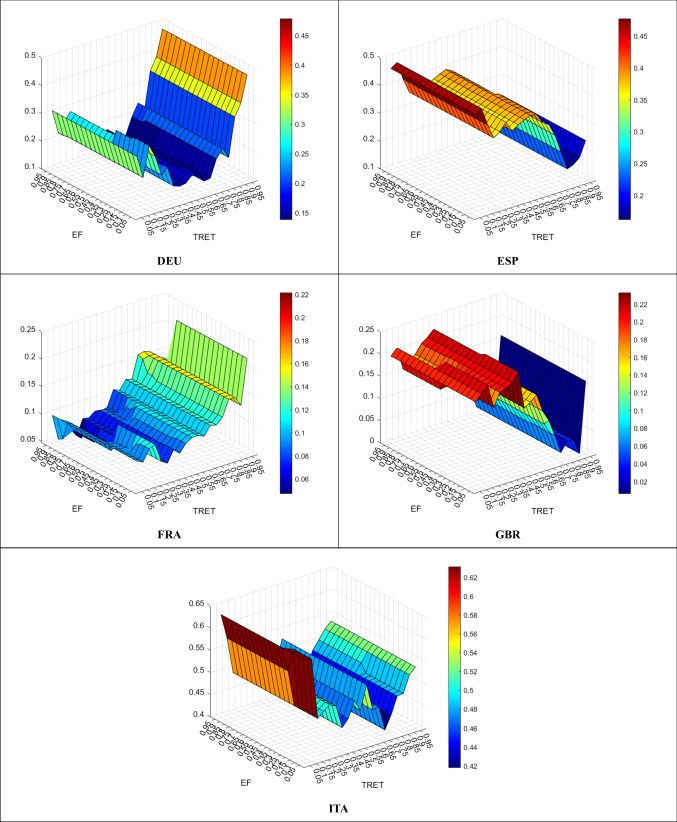
TRET Effect on EF

Figure 4 presents the effect of TRET on EF.

In all EU5 countries, TRET has an increasing effect on EF. In other words, TRET is not effective in curbing environmental pollution in all countries. At higher quantiles, TRET has a powerful stimulating effect on EF in DEU, FRA, and GBR, whereas the increasing effect is relatively horizontal in ITA and relatively lower in ESP. So, no EU5 countries can benefit from TRET in declining environmental pollution.

**Fig. 5:**
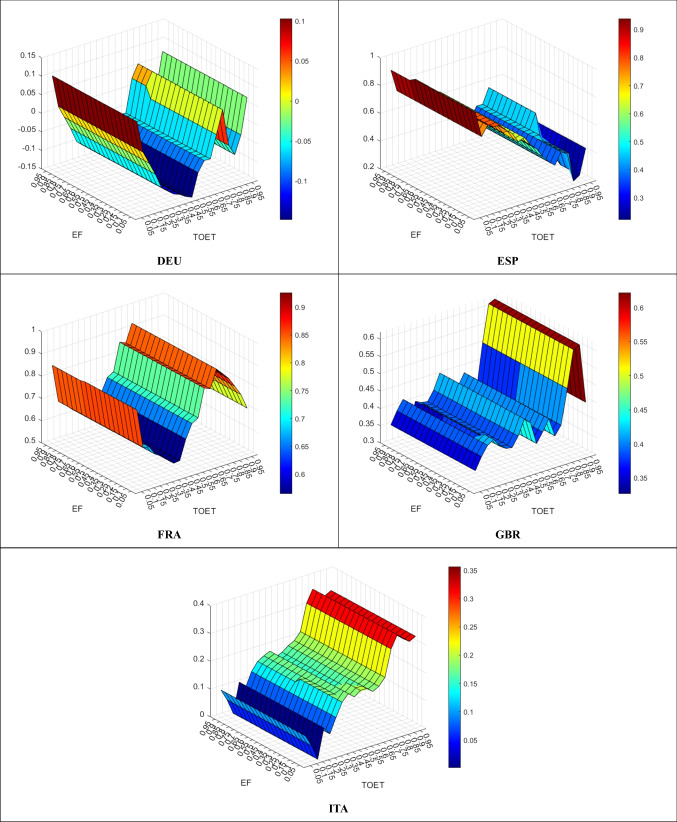
TOET Empact on EF

Figure 5 demonstrates the effect of TOET on EF.

In DEU, TOET has a curbing effect on EF across all quantiles except for some lower (0.05–0.10) and higher (0.70–0.75, 0.95) ones. On the other hand, in all remaining countries, TOET has an increasing effect on EF. At higher quantiles, the effect of ENET is highly increasing in ITA, weakly increasing in ESP, and relatively horizontal in both FRA and GBR:

Overall, while TOET is partially effective on environmental pollution in DEU, other countries cannot benefit from TOET in declining environmental pollution.

### Robustness

Finally, the QR method is performed to validate the robustness of the outcomes. Detailed comparison of QQ and QR methods are given in Annexes 1, 2, and 3, and a correlation summary between QQ and QR methods is presented in Table 5.

**Table 5:** Correlations of the QQ and QR methods

Variable	DEU	ESP	FRA	GBR	ITA
ENET on EF	99.93	99.67	99.92	99.99	99.92
TRET on EF	99.73	99.97	98.33	99.37	99.69
TOET on EF	99.98	99.42	99.99	99.74	99.95

According to Table 5, both QQ and QR methods’ outcomes are in high consistency, which is around 99.99%. Hence, the outcomes validate the robustness. So, the outcomes can be used to argue various policy options for EU5 countries.

## Conclusion, policy endeavors, and further research

### Conclusion

This research presents a comprehensive investigation of the effect of ET at disaggregated and aggregated levels on EF in EU5 countries. In this context, the study uses the most recent up-to-date dataset between 1995/Q1 and 2021/Q4 and applies novel nonlinear quantile-based approaches. Differently from the studies in the current literature, the study uses both disaggregated and aggregated level ET data together, considers EF as the more comprehensive environmental pollution indicator with regard to CO_2_ emissions, and applies nonlinear quantile-based approaches for the analysis across various quantiles.

The outcomes reveal that ET has a nonlinear effect on EF in the countries analyzed. Specifically, energy-related ET has only a declining effect at lower and middle quantiles in Germany and at lower quantiles in Italy. Also, transport-related ET is not effective on EF in any country, which means that it does not have a curbing effect. Besides, total ET has a decreasing effect in only Germany.

Overall, the study determines the nonlinear effect of ET on EF in the countries, whereas its power effect differentiates based on countries, quantiles, and types of ET in curbing environmental pollution. While the outcomes obtained are generally in line with the pre-expectations, however, they differentiate by presenting more comprehensive outcomes at both disaggregated and aggregated levels of ET.

### Policy endeavors

The outcomes mainly reveal the nonlinear effect of ET on EF across quantiles. Also, the effects of ET on EF vary according to countries, quantiles, and types of ET. Hence, a variety of policy endeavors can be discussed by relying on the outcomes.

EU5 countries should take recent empirical outcomes into account in re-formulating their ET practices. So, they can continue to apply ET practices in the areas, where they curb EF, as well as try to strengthen ET practices in some areas, where they do not provide a decline in EF. Hence, EU5 countries can benefit from ET in curbing environmental pollution. Hence, they can benefit from ET practices in achieving environment-related SDGs (i.e., SDG-7 and SDG-13).

Considering that the ET effect on EF differs based on countries, quantiles, and ET types (i.e., energy, transport, and total), EU5 countries should apply a continuous approach to monitor the ET effect over time and the areas, whereas ET has been applied (e.g., energy, transport, and total). Besides, EU5 countries should care about the changing ET effect on EF. For this reason, taking timely manner corrective actions is highly important to make inefficient ET practices effective in curbing EF.

It can be possible to argue that EU5 countries should include nonlinear approaches in their policy-making processes. Otherwise, EU5 policymakers may decide on the wrong points, which can be harmful to the main aims of ET practices and curbing efforts in environmental pollution.

Finally, each EU5 country should take into account its cases. That is why because the outcomes reveal that energy-related ET can be effective in only Germany and Italy, transport-related ET has no curbing effect on EF in any country, and total ET is only beneficial in Germany as well. So, restructuring ET practices to decline environmental pollution is highly required for EU5 countries. Otherwise, except for some of them (Germany and Italy), the EU5 countries cannot benefit from ET practices in declining environmental pollution.

### Further research directions

This study analyzes the environmental tax effect on ecological footprint in EU5 countries by following up a comprehensive approach. But the study is not free from some limitations that can be completed by future research. The study analyzes EU5 countries. Hence, future research can include other countries as well as country groups. Besides, new studies can make a disaggregated level analysis of ecological footprint by using sectoral data. Moreover, new studies can use much higher frequency data, if it becomes available. Furthermore, new studies can evaluated to consider tax autonomies in their empirical modelling. Also, recently emerged econometric approaches can be used in new studies. Hence, the literature can be extended further.

## Figures and Tables

**Annex 1:**
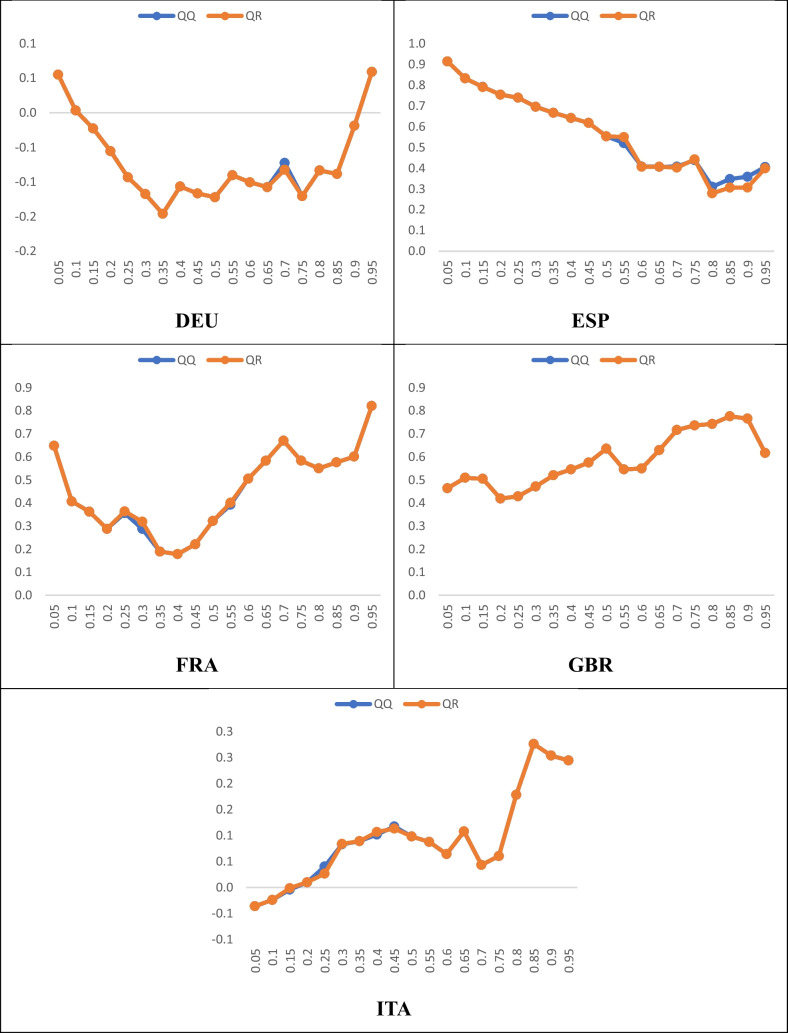
ENET effect on EF

**Annex 2:**
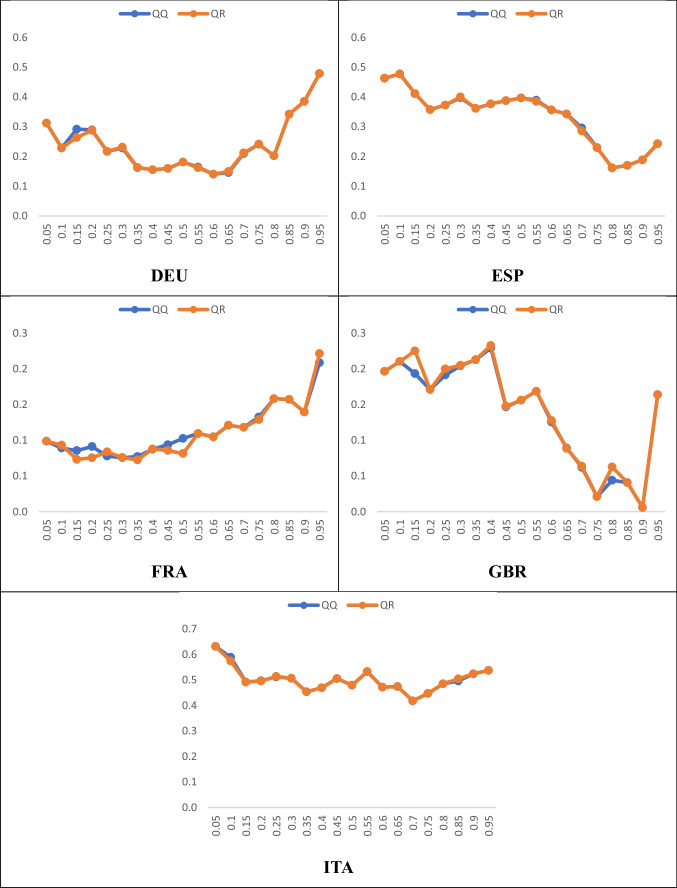
TRET effect on EF

**Annex 3:**
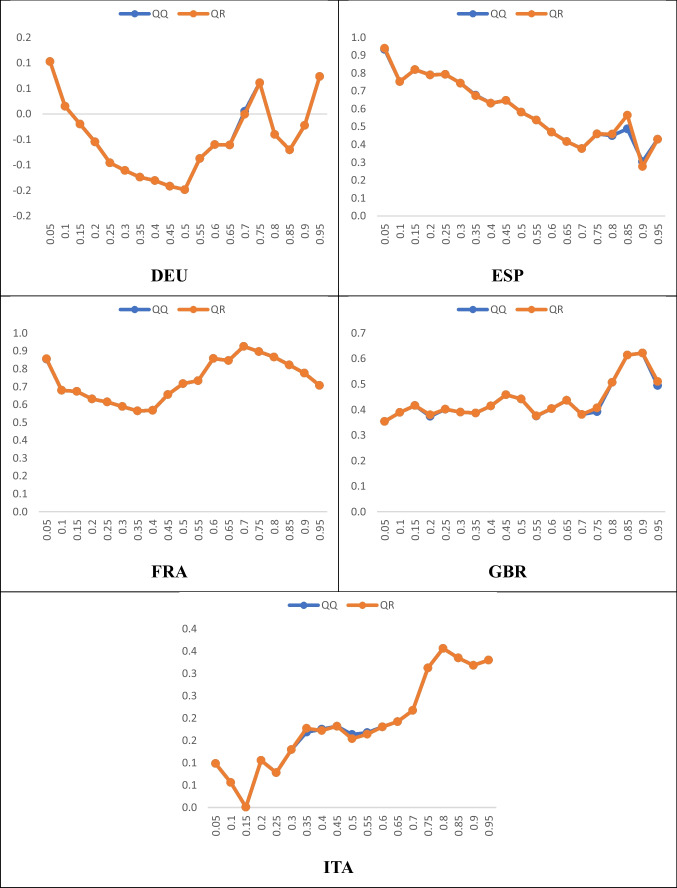
TOET effect on EF

## Data Availability

Data will be made available on request.

## Acronyms

BDS: Broock, Scheinkman, Dechert, and LeBaron
CO_2_: Carbon dioxide
ET: Environmental tax
EU5: European Union Five
GFN: Global Footprint Network
GHA: Global hectares
QQ: Quantile-on-quantile regression
QR: Quantile regression
SDGs: Sustainable development goals

## Dependent variable

EF: Ecological footprint

## Independent variables

ENET: Energy-related ET
TRET: Transport-related ET
TOET: Total ET

## Analysis scope (EU5 countries)

DEU: Germany
ESP: Spain
FRA: France
GBR: Great Britain
ITA: Italy
